# Functionalized Scintillating Nanotubes for Simultaneous Radio- and Photodynamic Therapy of Cancer

**DOI:** 10.1021/acsami.1c02504

**Published:** 2021-03-15

**Authors:** Irene Villa, Chiara Villa, Roberta Crapanzano, Valeria Secchi, Massimo Tawfilas, Elena Trombetta, Laura Porretti, Andrea Brambilla, Marcello Campione, Yvan Torrente, Anna Vedda, Angelo Monguzzi

**Affiliations:** † Dipartimento di Scienza dei Materiali, Università degli Studi Milano-Bicocca, via R. Cozzi 55, 20125 Milano, Italy; ‡ Stem Cell Laboratory, Department of Pathophysiology and Transplantation, Università degli Studi di Milano, Fondazione IRCCS Ca’ Granda Ospedale Maggiore Policlinico, Centro Dino Ferrari, via F. Sforza 35, 20122 Milan, Italy; § Dipartimento di Scienze dell’Ambiente e della Terra, Università degli Studi Milano-Bicocca, Piazza della Scienza, 20126 Milano, Italy; ∥ Servizio di Citofluorimetria, Laboratorio Analisi, Fondazione IRCCS Ca’ Granda Ospedale Maggiore Policlinico, via F. Sforza 35, 20122 Milan, Italy

**Keywords:** nanomaterials, radiotherapy, singlet oxygen, photodynamic therapy, scintillating nanoparticles

## Abstract

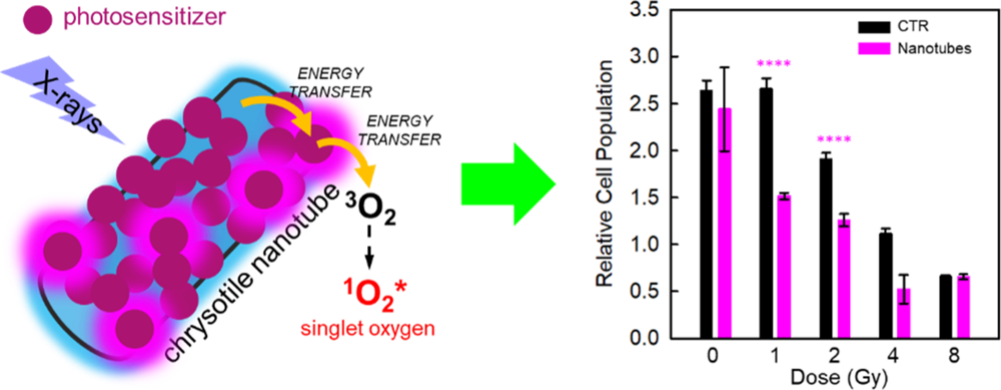

As a model radio-photodynamic therapy (RPDT) agent, we developed a multicomponent nanomaterial by anchoring conjugated chromophores on the surface of scintillating chrysotile nanotubes. Its ultimate composition makes the system a scintillation-activated photosensitizer for the singlet oxygen production. This nanomaterial shows a remarkable ability to enhance the production of singlet oxygen in an aqueous environment, under X-ray irradiation, boosting its production by almost 1 order of magnitude. Its efficiency as a coadjutant for radiotherapy has been tested *in vitro*, showing a striking efficacy in enhancing both the prompt cytotoxicity of the ionizing radiation and the long-term cytotoxicity given by radiation-activated apoptosis. Notably, the beneficial activity of the RPDT agent is prominent at low levels of delivered doses comparable to the one employed in clinical treatments. This opens the possibility of effectively reducing the therapy exposure and consequently undesired collateral effects due to prolonged exposure of patients to high-energy radiation.

## Introduction

Over the past few years, biomedical science has recognized the crucial role that nanotechnology can play in the field thanks to the development and use of nanoparticles in theranostics, which allows a deeper investigation of biological processes, faster diagnosis of diseases, accurate monitoring of specific injured tissues or organs, and, importantly, the improvement of some traditional therapeutic treatments.^1−5^ Due to their benefits with respect to larger systems, such as a high surface-to-volume ratio; facile surface functionalization; and tailorable optical, magnetic, and structural properties crucial for the adaptability to satisfy specific targets, nanomaterials are indeed ideal carriers for chemo- and phototherapeutic agents or radiosensitizers across several physiological barriers.^6,7^ Therefore, nowadays, a plethora of nanoscale materials, such as metallic and semiconductor nanoparticles, fluorites, and metal/lanthanide oxides, as well as organic and hybrid systems, are successfully exploited in advanced diagnostic and imaging techniques or innovative therapeutic approaches against cancer and other deadly diseases,^8−11^ as demonstrated by the more and more increasing number of nanosystems approved by the Food and Drug Administration (FDA) agency.^8,12^

In particular, biomedicine is moving toward the use of radioluminescent nanoparticles, that is, nanoscintillators, which are able to absorb and convert the ionizing radiation (X- or γ-rays) into a large number of UV/visible (UV/vis) photons exploitable to boost the efficacy of diagnosis routes, in nuclear medicine for preclinical mapping and intraoperative imaging and radiation dosimetry, and as coadjutants in oncological therapies.^13−16^ The search for innovative therapies overtaking state-of-the-art oncological treatments is challenging. Conventional cancer treatment options—chemotherapy, radiotherapy (RT), and surgery—are still associated with systemic side effects, disease recurrence, and drug/radio resistance of malignant cells. In particular, ionizing radiation is used in approximately 50% of all cancer treatments to stop the rapid proliferation of cancer cells directly by damaging their DNA and by thermal shock or indirectly by forming cytotoxic free radicals, that is, reactive oxygen species (ROS) such as hydroxyl radicals and singlet oxygen, upon interaction with the intracellular aqueous environment.^17,18^ However, RT is limited by the maximum radiation dose that can be given to a tumor mass without incurring significant injuries to the adjacent tissues or organs.^19^ In order to maximize the therapeutic efficacy and, possibly, to reduce the required X-ray dose, thus limiting the collateral damage of surrounding healthy tissues, high-atomic-number and dense nanoparticles are currently evaluated as coadjutants. They are indeed potential safe and effective means for the treatment of radiosensitive and radioresistant tumors thanks to the enhanced interaction of the incorporated heavy elements with the high-energy radiation. The increased probability of radiation interactions in the presence of tumor-targeting nanoparticles results in the release of a large number of secondary free carriers that can enhance the local damage without affecting the surrounding healthy tissue.^20,21^ A step forward in the development of alternative oncological treatments can be made by synergistically coupling the well-assessed RT with complementary methods, such as the photodynamic therapy (PDT). PDT is considered as a clinically deployed efficient and non-invasive alternative to surgery and to the current oncological therapies due to spatial specificity larger than that of RT and chemotherapy and being not subjected to dose limitation. PDT is based on the cytotoxic effects originating when biocompatible photosensitizers (PSs) are photoexcited, producing ROS and thus inducing the cell death through oxidative damage of cellular membranes.^22−24^ Unfortunately, the PSs approved for routine PDT treatment require UV/vis light to be activated. In this spectral region, the human tissue transparency is low, thus making PDT ineffective for tumors seated at depths larger than 1 cm.^25,26^ Considering that for deep-tissue treatment, the advantages of the use of high-penetrating ionizing radiation for energy transport are unparalleled, the realization of an effective PDT-enhanced RT will allow to overcome simultaneously the PDT depth restriction and the high radiation doses/low selectivity drawbacks of RT through the synergistic combination of both healing methods.^27,28^

In general, this strategy accounts for the employment of a nanoscintillator structurally coupled to a PS therapeutic agent, whose electronic energy levels are resonant with the nanoscintillator emission energy. When the ionizing radiation excites the nanoscintillator, instead of activating its luminescence, the absorbed energy is transferred to the PS through an energy-transfer process. Therefore, the sensitization of the ROS species is activated in deep tissues, realizing a synchronous and colocalized radio- and photodynamic (from here, radio-photodynamic, RPD) therapy (RPDT). In this framework, many inorganic nanomaterials like oxides, fluorides, silica-based nanostructures, and semiconductor nanocrystals have been combined with organic PSs toward RPDT applications; also, metal–organic frameworks containing heavy metals and PS molecules in their structures have been proposed as RPDT agents.^16,18,29−35^ Remarkably, several works have demonstrated an important synergetic effect of the synchronous use of RT and PDT, further supporting the development of this method.^19,35^ The debate on the potential mechanisms of RPDT that determine the radiation dose enhancement in the vicinity of the malignancy due to the presence of high-*Z*/dense elements is open and lively and devoted to the comprehension of the complex correlations between the increase of the ionizing radiation interaction cross-section, the effective energy release within the nanomaterial and in its biological surrounding, and the phototoxic effect of the PSs incorporated in or grafted on nanoscintillators. Despite the experimental evidence, no unique explanation exists that justifies the RPDT efficacy, that is, whether the dominant therapeutic effect is due to the presence of high-*Z* elements allowing for an enhancement of radiation dose/energy deposition ratio in the malignant tissues or the establishment of a tradeoff between PDT and RT resulting in a synergistic enhancement of the efficiency in killing the tumor cells or in the overall reduction of the total dose to the patient.

**Figure 1:**
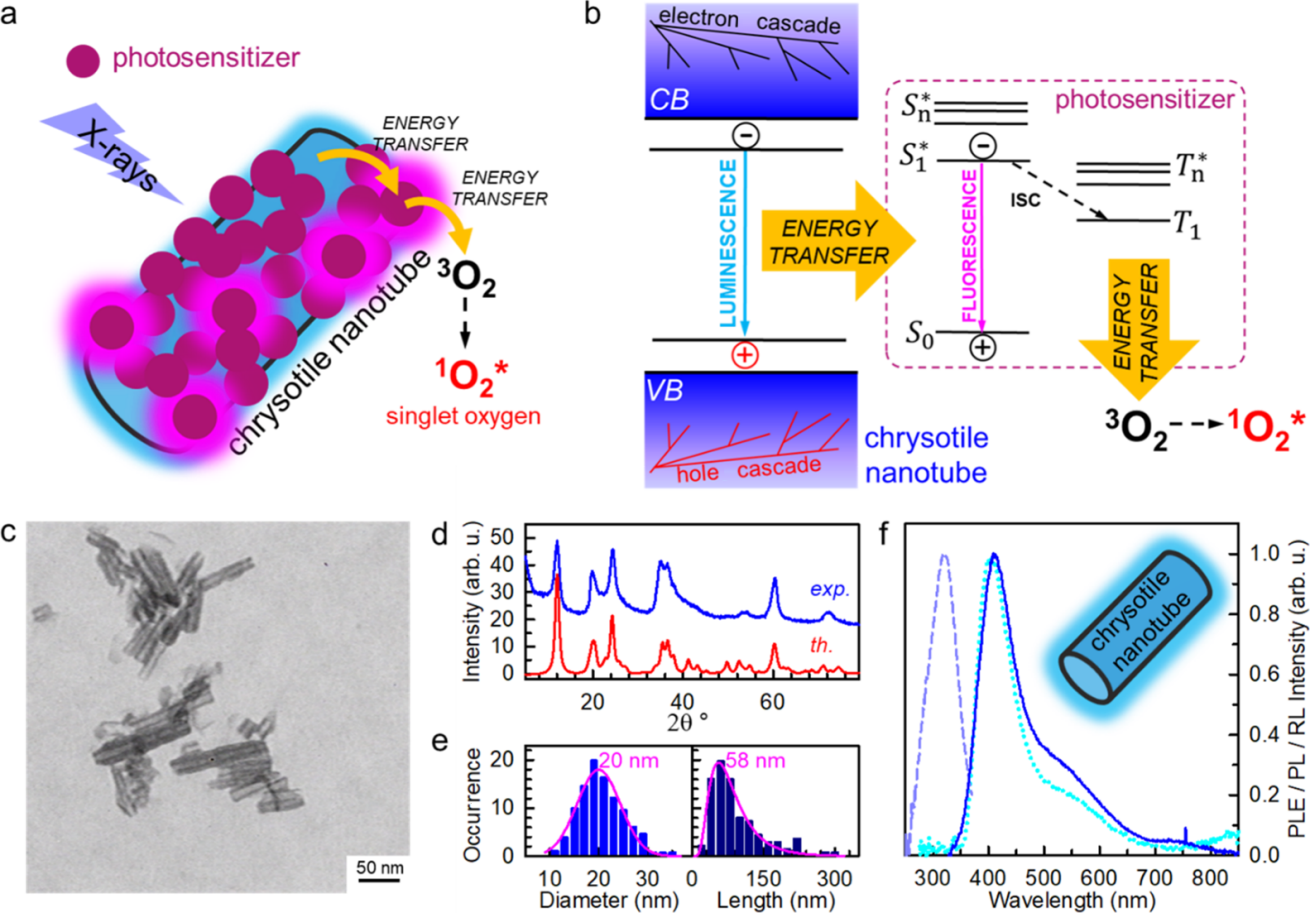
(a) Sketch of a surface-functionalized scintillating chrysotile NT decorated with a singlet oxygen PS. Glowing dots highlight luminescent PSs. (b) Outline of the photophysical process involved in the sensitization of singlet oxygen (^1^O_2_^*^) production upon irradiation with X-rays. Free electrons and holes generated by interaction with the ionizing radiation in the NT localize at an emissive state that transfers its energy to the PS molecules on the surface promoting their excited singlet state (S_1_^*^). Upon ISC, the energy is transferred from the PS triplet to the dispersed molecular oxygen in triplet ground-state ^3^O_2_, which is promoted to its excited singlet state. (c) TEM images of bare NTs. (d) PXRD patterns of a reference chrysotile sample (th.) and as-synthesized bare NTs (exp.). (e) Distribution of lengths and diameters obtained by the analysis of TEM images. (f) PL (dots), PLE (dash), and RL (solid line) spectra of bare NTs under 320 nm excitation and soft X-ray exposure, respectively.

In this work, as illustrated in Figure 1a, we developed a multicomponent RPDT agent by coupling scintillating chrysotile nanotubes (NTs) with conjugated chromophores anchored on their surfaces as a PS for the singlet oxygen. Upon irradiation with ionizing radiation, the PS is activated by resonant energy transfer from the luminescent NT. A subsequent energy transfer to ground-state oxygen molecules in the aqueous environment promotes the creation of the singlet oxygen species. The singlet oxygen sensitization capability and the cytotoxic attitude of each component of the system have been quantitatively compared to obtain the guidelines for the design of the most efficient RDPT agent. The optimized nanosystem shows a remarkable ability to enhance the production of singlet oxygen in the aqueous environment under X-ray irradiation, boosting its production by almost an order of magnitude. Its efficiency as a coadjutant for RT has been tested *in vitro* on the human glioblastoma primary cell line (U-87), showing a striking efficacy in enhancing by almost an order of magnitude the prompt cytotoxicity of the ionizing radiation at low dose values delivered in human therapies, as well as a remarkable long-term effect leading to loss of clonogenic capacity of the tumoral cells.^18^

## Results and Discussion

### Material Design and Optimization

Figure 1b sketches out the photophysical processes involved in the singlet oxygen production in the presence of functionalized scintillating NTs upon irradiation with X-rays. After the interaction with ionizing radiation, several steps are involved before the generation of the ROS. The interaction of a high-energy beam with the nanoscintillator promotes the generation of high-energy free charges, electrons and holes, a fraction of which can diffuse through the NT toward luminescence centers (*vide infra*) where they can recombine radiatively.^36^ In proximity of a suitable energy acceptor, such as the grafted PS molecules, the energy stored in the NT can be therefore transferred by energy-transfer mechanisms. In our case, the energy transfer from the nanoscintillator promotes the first excited singlet state of the PS. This can recombine radiatively, producing fluorescence, or non-radiatively by intersystem crossing (ISC) toward its triplet state, from which a subsequent non-radiative energy transfer to the molecular oxygen dispersed in the cellular environment sensitizes the population of the cytotoxic singlet oxygen.^37^ We employ synthetic chrysotile NTs as nanoscintillators because according to the literature, these nanostructures are highly biocompatible particles that can be prepared in aqueous solution under hydrothermal conditions in the presence of Mg and Si precursors.^38^ With the chemical formula Mg_3_Si_2_O_5_(OH)_4_, chrysotile is a layered silicate having alternating layers of silica, SiO_2_, and brucite, Mg(OH)_2_ (Figure 1d),^39^ which are wrapped up giving rise to a tubular habit (Figure 1c). By tuning the reaction time, the size distribution of the NTs can be controlled to a certain extent. Our aim was to obtain a particle size as close as possible to the optimum value for cell internalization, that is, around 50–60 nm.^40^ As shown in Figure 1e, while the diameters follow closely a normal distribution centered at 20 nm, the lengths obtained follow a log-normal distribution with a maximum at 58 nm. This behavior is indicative of a homogeneous mechanism for particle nucleation.^41^ The external surface of the NTs is brucitic. This confers a basic behavior to the surface which, under a mild acidic environment, tends to concentrate Mg^2+^ ions, assuming a positive ζ-potential.^42^ This property allows to bind to the surface a number of anionic chemical species, including our selected PS dyes. The luminescence properties of the bare NTs are explored under optical and X-ray excitation (Figure 1f). Under UV excitation, the NTs show blue photoluminescence (PL) peaked at 420 nm, with an excitation maximum in the PL excitation (PLE) spectrum at 320 nm. Upon irradiation with soft X-rays, the NTs show a broad emission, ranging from the near UV up to the red, dominated by a blue radioluminescence (RL) component that well matches the PL profile, thus suggesting that the same recombination centers are responsible for the NT luminescence under both selective (PL) and non-selective (RL) excitation. Briefly, the chrysotile luminescence properties lying in the 450–550 nm range are associated to the presence of intrinsic defects within the lattice as well as the formation of self-trapped excitons. Other contaminants, such as other minerals and trace metals, can act as luminescence centers themselves or can create optically active centers giving rise to the chrysotile emissions. A detailed discussion on the origin of emission components is reported in the Supporting Information.

**Figure 2:**
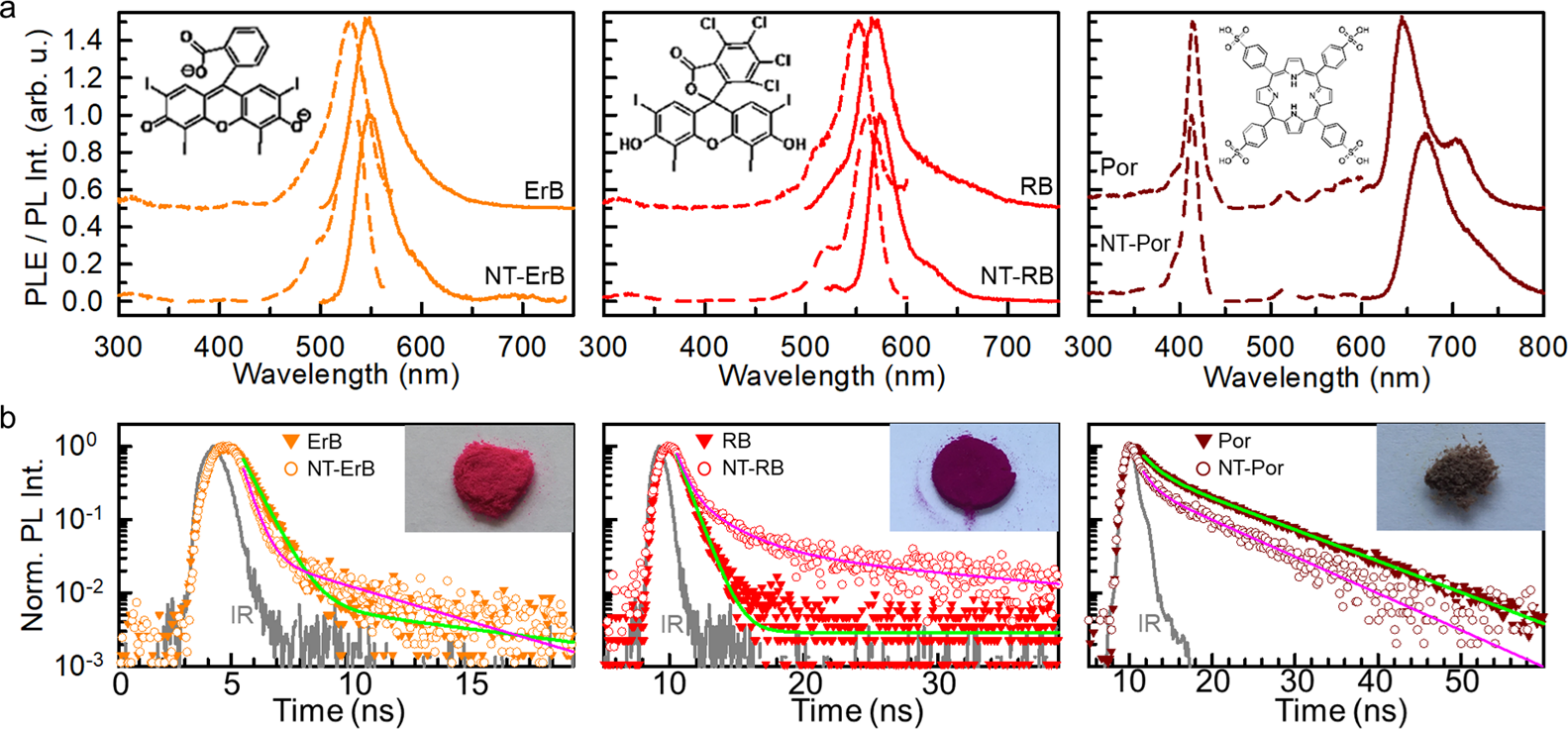
(a) PL (solid lines) and PLE (dashed lines) spectra of the PSs ErB, RB, and H_2_TPPS^4–^ porphyrin (Por) in solution and attached on the NT surface (NT-ErB, NT-RB, and NT-Por, respectively). PL spectra have been recorded under 510 nm (ErB, NT-ErB, RB, and NT-RB) and 405 nm (Por) excitation. Decorated NTs have been dispersed in phosphate-buffered saline (PBS) to mimic the cellular environment. The molecular structure of the tested PSs is reported in the inset. (b) Fluorescence decay in time of PSs in the diluted PBS solution and decorated NTs dispersed in PBS. Time-resolved PL spectra are recorded at the maximum of the PL spectrum under pulsed excitation at 510 nm for ErB, NT-ErB, and NT-RB, 405 nm NT for NT-Por. Solid lines are the fit of the emission intensity decay with the multiexponential function. Insets are digital pictures of the as-synthetized functionalized NTs.

In order to find the most performing combination, we evaluated a series of conjugated chromophore systems known to be efficient PSs. In particular, we tested Erythrosine B (ErB), Rose Bengal (RB), and meso-tetra(4-sulfonatophenyl)porphyrin (H_2_TPPS^4–^). Their molecular structures are reported as insets in Figure 2A. All of them show a good singlet oxygen generation yield >60% upon photoexcitation in diluted solution,^24,43^ and, importantly, their ground-state absorption (Figure S2) is resonant with the NT emission energy, thus enabling energy transfer once bound onto a NT. According to previous results, the synthesis condition is initially set to obtain a full coverage of the NT surface.^38^ The electronic properties of anchored dyes, pivotal for efficient singlet oxygen sensitization, are monitored by means of continuous-wave (*cw*) and time-resolved PL spectroscopy. Figure 2a is a comparison between the emission and excitation properties of dyes in diluted solution and grafted on NTs. The PL shape of grafted ErB and RB is identical to that one in diluted solution, while the emission profile of anchored porphyrins is slightly red-shifted with respect the solution case in agreement with the tendency of this dye to form J-type aggregates.^44,45^ Nevertheless, the PLE spectra and absorption (Figure S2) of grafted dyes match perfectly the one of isolated dyes, demonstrating that the resonance with the NT emission required for energy transfer is maintained. The analysis of the PL decays in Figure 2b gives further insight into the excited-state properties of anchored dyes. The parameters of the fit functions used to analyze the data are reported in the Supporting Information, Table T2. For all dyes, in diluted solution, the PL intensity decays substantially as a single exponential function with the characteristic decay time τ = 0.7, 0.9, and 10.2 ns for ErB, RB, and porphyrin, respectively. Due to the presence of a minor fraction of J-aggregates that act as quenchers,^45^ we note only a slight acceleration of the PL intensity decay of porphyrin-functionalized NTs with respect to the one of the isolated porphyrin, which emits with a characteristic time of ∼10 ns. On the contrary, the recombination dynamics of ErB and RB is significantly different with respect to the diluted solution case. For ErB, the decay shows a bi-exponential behavior. Most of the excited states (93%) decay faster than the diluted solution, while the second component shows a longer lifetime. This finding hints both the presence of competitive deactivation pathways and the presence of alternative radiative recombination centers, including aggregates and excimer species that can originate from the interaction of close-packed chromophores on the NT surfaces. For RB, the decay shows even more marked multiexponential behavior, which hints the presence of a broad distribution of emitters. The detailed analysis of intermolecular interactions on NT surfaces to shed light on these aspects would require further dedicated investigations, but, in general, these results show that the excited-state recombination dynamics of ErB and Bengal Rose is heavily affected by their arrangement on the NT surface, with potential detrimental consequences on their efficacy as PSs (*vide infra*).

**Figure 3:**
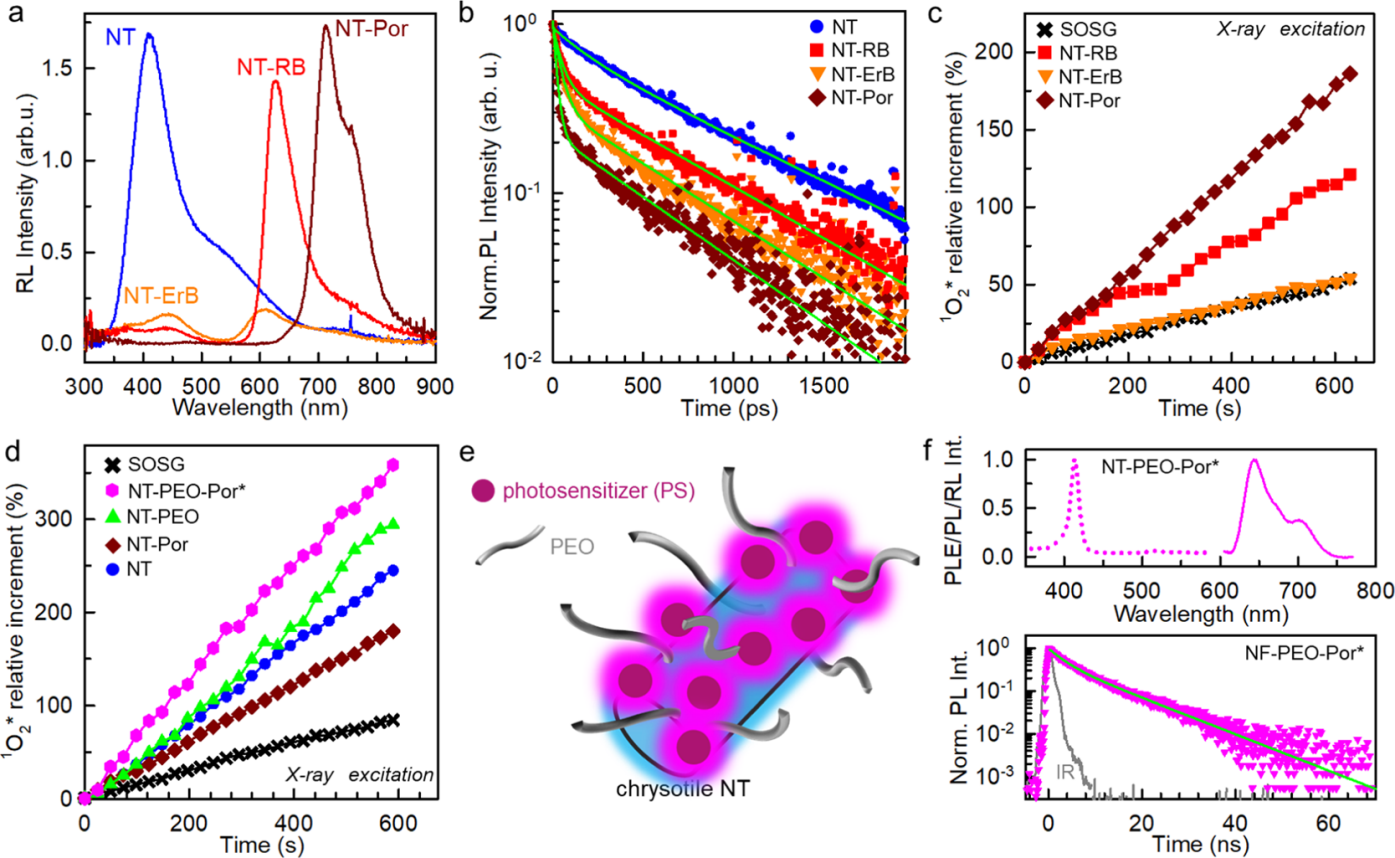
(a) RL spectra of bare NTs (NT) and NTs functionalized with ErB (NT-ErB), RB (NT-RB), and the H_2_TPPS^4–^ porphyrin (NT-Por). (b) Time-resolved PL spectrum at 410 nm of bare and functionalized NTs. Solid lines are the fit of the experimental data with a bi-exponential function used to calculate the efficiency of the energy transfer from NTs to molecules grafted on surfaces. (c) Measurement of the relative singlet oxygen concentration in PBS dispersions of dye-functionalized NTs as a function of time under X-ray exposure. (d) Measurement of the relative singlet oxygen concentration in PBS dispersions of bare NTs, porphyrin-functionalized NTs (NT-Por) and functionalized NTs stabilized with PEO (NT-PEO, NT-PEO-Por, and NT-PEO-Por*) as a function of time under soft X-ray exposure. The NT-PEO-Por* sample has been prepared with a dye dilution of 1:50 with respect the NT-Por sample. (e) Sketch of the NT-PEO-Por* composition. (f) PLE, PL, and time-resolved PL spectra of the NT-PEO-Por* sample dispersed in PBS.

The luminescence properties of functionalized NTs have been studied under irradiation with soft X-rays. As expected, the conjugated PSs alone show an RL intensity about 3 order of magnitudes weaker than that of functionalized NTs due to their low density and atomic number *Z* (Figure S3). Figure 3a reports the RL spectrum of powder samples before and after the functionalization (see Methods). The blue emission of bare NTs is quenched upon functionalization, while dominant emission bands appear at lower energies matching the fluorescence profile of each dye. These findings suggest that a portion of energy deposited in the NTs is successfully transferred to dyes activating their fluorescence and, therefore, the sensitization of singlet oxygen. The energy-transfer efficiency (ϕ_ET_) is initially estimated from the ratio between the integrated intensities of the NT blue emission with (*I*_PS_) and without (*I*_0_) functionalization as^46^
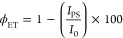
1

Equation 1 returns a yield of 90% for energy transfer toward ErB, 93% toward RB, and 99% toward porphyrin. To discriminate the role of non-radiative energy transfer, this latter has been further investigated by time-resolved experiments on diluted NT dispersions. Figure 3b shows the PL decay of the NT PL at 420 nm under pulsed excitation at 360 nm. The PL intensity of bare NTs decays with a multiexponential function, most probably due to energy migration toward defects or other quenching centers within their structure, with a calculated average lifetime of τ_NT_ = 670 ps (Supporting Information and Table T3) and a PL quantum efficiency <1% (Methods), which confirms the occurrence of quenching mechanisms for the NT luminescence. Upon functionalization, the PL decay assumes a bi-exponential character. A remarkable acceleration appears at short times in the first 200 ps, which mirrors a fast, non-radiative energy transfer from the excited NT to the grafted dye molecules, as expected due to their close proximity and their electronic states. Specifically, the fast component has characteristic lifetimes of τ_fast_ = 49, 45, and 10 ps for ErB, RB, and porphyrin, respectively. Therefore, we can estimate the non-radiative energy-transfer yield as^46^
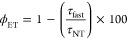
2which results in an efficiency as large as ∼93% for ErB and RB and ∼100% for porphyrins. At times >200 ps, the spectra of functionalized NTs show a second component with a characteristic decay time very close to the one of the bare NTs that is observed in all the compositions. We ascribe this slow component to a fraction of NTs where the energy transfer is negligible, probably due to partial coverage of the structures or due to the presence of aggregated species, as hinted by the data in Figure 2b, which are not able to receive energy from the nanoscintillator. The net non-radiative energy-transfer yield ϕ̅_ET_ for the NT ensemble has therefore been calculated as
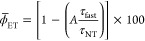
3where *A* is the relative weight of the fast component in the time-resolved PL spectrum, thus resulting in ϕ̅_ET_ = 47, 65, and 85% for ErB, RB, and porphyrin, respectively (Supporting Information and Table T4).^46^

### Singlet Oxygen Generation Performance

Considering the measured energy-transfer yields, the luminescence intensity under X-ray excitation, and the excited-state properties investigated by time-resolved experiments, the NTs functionalized with porphyrins appear to be the best candidate as an efficient RPDT agent. This conclusion is further supported by the direct measurement of the relative singlet oxygen production efficiency under X-ray exposure. Figure 3c reports the evolution of the singlet oxygen concentration in the PBS dispersion for the functionalized NT series, which has been monitored *in situ* by using the Singlet Oxygen Sensor Green (SOSG, see Methods) as an optical probe (Figures S5 and S10).^47^ After 600 s of irradiation, in the presence of the porphyrin-functionalized NTs, we observe a 4 times enhancement of the singlet oxygen concentration. Using RB, the amount of ROS species is only doubled, while no difference can be detected using ErB. Therefore, the porphyrin has been selected as the designated PS for further experiments.

The data presented demonstrate that the presence of dense NTs to which photosensitizers are anchored is beneficial for the production of singlet oxygen in the aqueous dispersion. The high-energy beam has indeed a first interaction with the material by the photoelectric effect or Compton scattering, and the created secondary charges can start travelling through the NT. They can recombine to create emissive states from which energy transfer to PSs occurs or even directly recombine on the grafted molecules, in both cases activating the singlet oxygen sensitization by dyes. However, it is worth to mention that the energy transferred from the high-energy beam and the secondary charges cannot be fully deposited into the nanoscintillators because of its small size in comparison with the travelling distance of the released energetic charges.^48^ The energy available to be shared between the nanoscintillator and the photosensitizers is indeed only a fraction of that of the first exciting beam, while the remaining part can activate directly the ROS production, for example, through an enhanced water radiolysis.^49^ Therefore, in order to confirm an effective RPDT performance of a multicomponent material, it is pivotal to evaluate the ROS-sensitization ability of each component of the system. Figure 3d shows the comparison of the singlet oxygen generation performance of bare and fully covered functionalized NT dispersion in PBS. The experiment shows that bare NTs already work as sensitizers for singlet oxygen, similar to other metal oxide nanostructures,^50,51^ thanks also to the catalytic effect of their surfaces in the production of ROS.^52^ Additionally, they work better than the functionalized case. We ascribe this peculiar effect to the fact that not all the adsorbed porphyrins can work as sensitizers. Indeed, both *cw* and time-resolved PL experiments demonstrated the presence of aggregates, which possess different electronic properties that can affect the sensitization of singlet oxygen. Specifically, porphyrins’ J-aggregates feature a red-shifted PL and negligible emission yield, thus limiting the energy transfer to oxygen and consequently the generation of excited singlet oxygen molecules. Also considering the diffusion of molecular excitons within the porphyrin layer to be trapped by the J-aggregate low energy state, it is not surprising that the effective sensitization performance of the system is lower than expected. Therefore, the full coverage of the NT surfaces by dyes could result in a lower singlet oxygen sensitization because surface interactions with the environment are partially hindered. Moreover, our results demonstrate that close-packed dyes do not show the same efficiency as single-molecule PSs, thus basically draining energy from NTs without efficiently supporting the ROS production.

In order to overcome this issue, we synthesized a new batch of NTs with a lower coverage level by using a 50 times lower initial concentration of porphyrin. In addition, the available free surface of the NTs has been exploited to improve the stability of functionalized NTs in aqueous environments by a subsequent decoration with mPEG2K-phosphate (PEO) as a stabilizer (the Methods and Figure S6).^53^ Surface modification with PEO is advantageous because it is FDA-approved and particularly low-priced. Moreover, it increases the *in vivo* circulation time because it prevents agglomeration of nanosized structures and opsonization from the immune system.^54−56^ The resulting multicomponent system (NT-PEO-Por*, Figures 3e, S6, and S7) shows a remarkably improved dispersion stability in PBS and, importantly, luminescence properties strictly similar to the one of the single porphyrin molecule, with an emission peaked at 670 nm and a PL lifetime of about 10 ns (Figure 3f; Supporting Information, Table T2 and Figures S8 and S9). These findings suggest that this composition maintains the PS properties even when grafted on the NT surface, and thus, an enhanced singlet oxygen sensitization ability is expected. The data in Figure 3c indicate that PEO-stabilized NTs, without dyes, already show an improvement with respect to the bare NT case, most probably due to the better colloidal dispersion achieved with PEO functionality that avoids sedimentation or aggregation and maximizes the interaction with the aqueous environment. Remarkably, stabilized NTs functionalized with porphyrins show an even better ability to sensitize the singlet oxygen species thanks to the synergistic contribution of a high-density material, which enhances the interaction with high-energy radiation, and the effective action of PSs activated by the nanoscintillators when grafted on its surfaces. The role of grafting is both to enhance energy transfer and to enhance favoporphyrin absorption of low-energy carriers surrounding the NTs generated by ionization events in the high-density material (Figure S11). In particular, with the addition of the optimized sensitizers NT-PEO-Por* (hexagons in Figure 3c), the relative concentration of singlet oxygen is increased by almost an order of magnitude in comparison to the amount generated spontaneously by the interaction of the ionizing radiation with the aqueous environment (crosses in Figure 3c), thus supporting their evaluation as RPDT agents in the biological environment.

**Figure 4:**
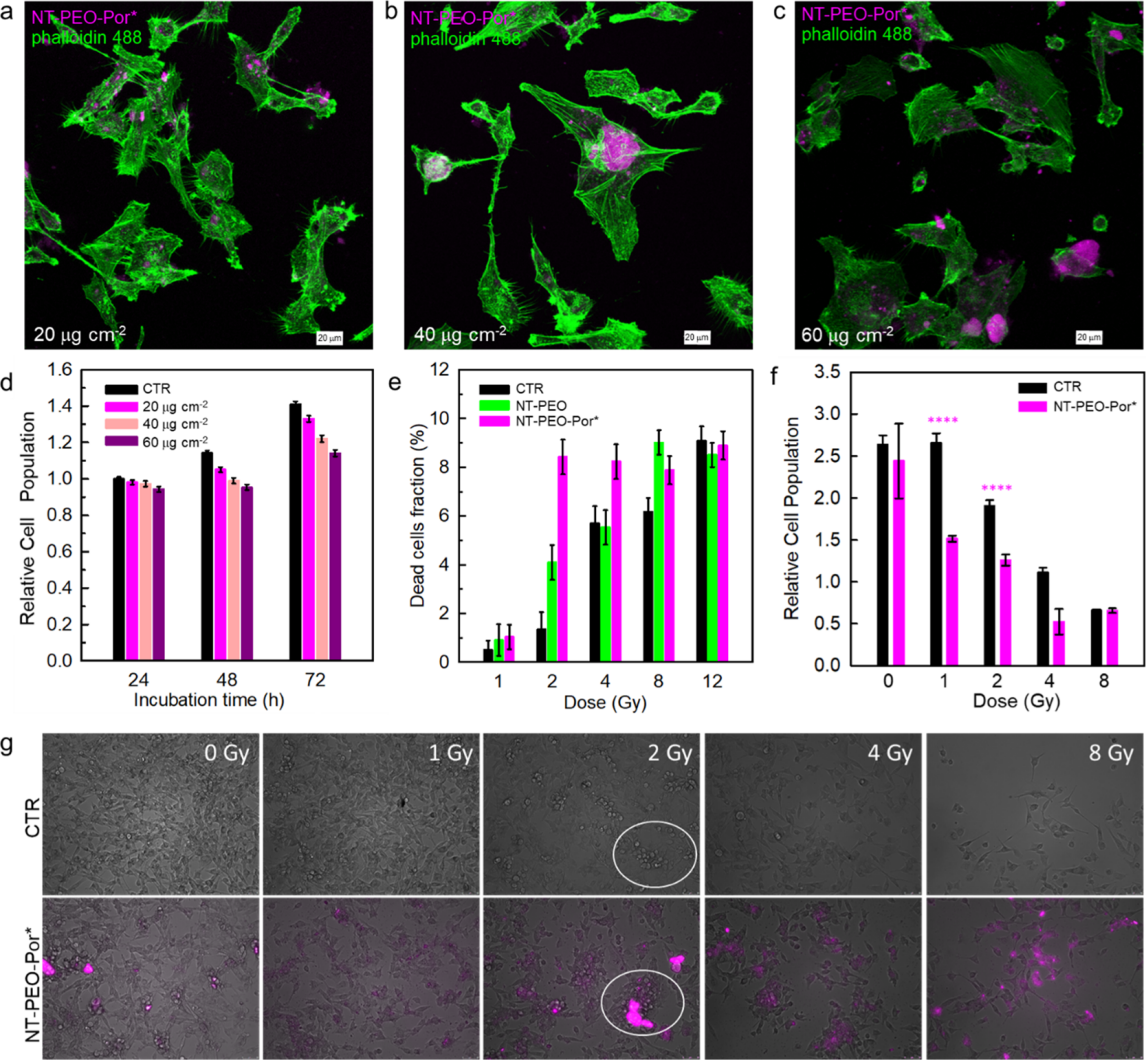
(a–c) Confocal fluorescence images of human glioblastoma cell line U-87 costained with phalloidin 488 for the F-actin filaments of the cytoskeleton (green) and with NTs functionalized with PEO and a 1:50 porphyrin concentration (NT-PEO-Por*, pink). (d) Evaluation of cell metabolic activity by the MTT test on U-87 cells stained with 20, 40, and 60 μg cm^–2^ of NT-PEO-Por*. (e) Relative fraction of dead cells estimated by the Trypan blue cell exclusion assay on U-87cells stained with 20 μg cm^–2^ of NT-PEO-Por* as a function of the nominal dose delivered. (f) Evaluation of cell metabolic activity by the MTT test on U-87 cells stained with 20 μg cm^–2^ of NT-PEO-Por* as a function of the nominal dose delivered. MTT assays and Trypan blue cell counting were performed in triplicate. Statistical analysis: two-way ANOVA, *P* < 0.0001 ****. Error bars are the standard deviations of the mean values calculated for five independent experiments. (g) Wide-field images of the U-87 cell culture without (top) and with (bottom) 20 μg cm^–2^ of NT-PEO-Por* stain as a function of the nominal delivered dose. The white circles highlight cellular debris and necrotic blebs that appear at irradiation doses >2 Gy.

The biocompatibility and RPDT activity of the NT-PEO-Por* multicomponent NTs are evaluated by means of well-established viability and cytotoxicity assays on the human glioblastoma primary line U-87 cells, which is a well-known reference system employed in the studies of glioma.^57^Figure 4a–c shows the confocal fluorescence imaging of cells stained with different concentrations of NT-PEO-Por*, 20, 40, and 60 μg cm^–2^, respectively, and phalloidin 488 for cytoskeletal F-actin staining and therefore confirms the NT uptake.^58^ In all samples, upon laser irradiation at 405 nm, the fluorescent NTs can be visualized as pink aggregates. However, it can be noticed that only at the lowest concentration of 20 mg cm^–2^, the NTs that entered cells appear quite well dispersed in the cytoplasm, while small aggregates are likely attached on the cellular membrane. Conversely, at higher concentrations, significantly big aggregates appear, which can represent a crucial stress factor for the cells. Indeed, the results of the viability test shown in Figure 4d indicate that using NT concentrations of 40 and 60 μg cm^–2^, the cellular proliferation is slowed down with respect to the unstained control culture, which is 15–20% more populated after 72 h of incubation. Therefore, the stain concentration of 20 mg cm^–2^ is chosen to perform any further experiment since no significant change (<5%) in the cell population is observed after the same incubation time. The RPDT effect of functionalized NTs is monitored by exposing the cell seeded onto tissue culture dishes to soft X-rays for different irradiation times in order to control the nominal dose delivered to the cells.

The sensitization of the radiation cytotoxicity in the stained cells is verified by two different assays. Figure 4e shows the results obtained with the Trypan blue cell exclusion assay, performed immediately after irradiation, which allows to directly compute the number of dead cells, stained with blue, by bright-field optical microscopy. In this case, we compared a sample stained with NT-PEO NTs, that is, without the porphyrinic functionality, with the most efficient singlet oxygen sensitizer NT-PEO-Por* tubes and a control sample of unstained cells. The sensitization of cytotoxic activity in the presence of NTs is evident in both the stained cultures. The NT-PEO staining already induces a good RT efficacy enhancement, increasing by 150% the number of dead cells with respect to the control sample when a 2 Gy dose is delivered. Remarkably, with the NT-PEO-Por* staining, the fraction of dead cells is enhanced by 500%. At 4 Gy of delivered doses, only the NT-PEO-Por* staining enables a further improvement of the cellular killing efficiency, while for larger doses, the direct destructive effect of the ionizing radiation dominates, without noticeable difference between stained and unstained cultures. These results are remarkable since they proof the synergistic effect of the coupling of dense nanoscintillators, which enhances the interaction with the high-energy radiation increasing the amount of cytotoxic dose released in the cellular environment, with an efficient singlet oxygen sensitizer activated by the nanoscintillator emission, thus sustaining the production of cytotoxic ROS and therefore further enhancing the therapeutic effect of the ionizing radiation. It is important to highlight that the presence of the RPDT agents brings about a significant enhancement, especially at low delivered doses between 1 and 2 Gy, that is, a range compatible with the currently applied clinical protocols. We also notice that in the presence of NTs, many cells appear directly affected by ionizing radiation-induced hyperthermia. This latter can cause a dose-dependent heat shock, leading to immediate cell necrosis characterized by plasma membrane swelling and rupture. Figure 4g depicts indeed the occurrence of cell debris and blebs in NT-PEO-Por*-stained U-87 cells after 24 h from X-ray exposure, with signs of cell membrane shrinking and contraction starting from a 1 Gy dose that may correlate with forthcoming cell death.^59^ This rapid mechanism of cell death is scarcely quantifiable since degraded membranes and debris are not attributable to an exact cell number, thus inducing some uncertainty to the Trypan assays. Therefore, we performed a control test using the MTT assay to measure the cellular metabolic activity as an indicator of cell viability, proliferation, and cytotoxicity.^60^ The histogram in Figure 4f depicts the result of the MTT assay obtained on U-87 cells stained with NT-PEO-Por*, which confirm the previously obtained results. As in the case of Trypan blue counting, thanks to presence of the RPDT agent, we observe indeed a net enhancement of the cytotoxic ability of the ionizing radiation, especially in the range of 1–4 Gy of delivered dose where the population of living cells is reduced with respect to the unstained reference system by 30–40%.

**Figure 5:**
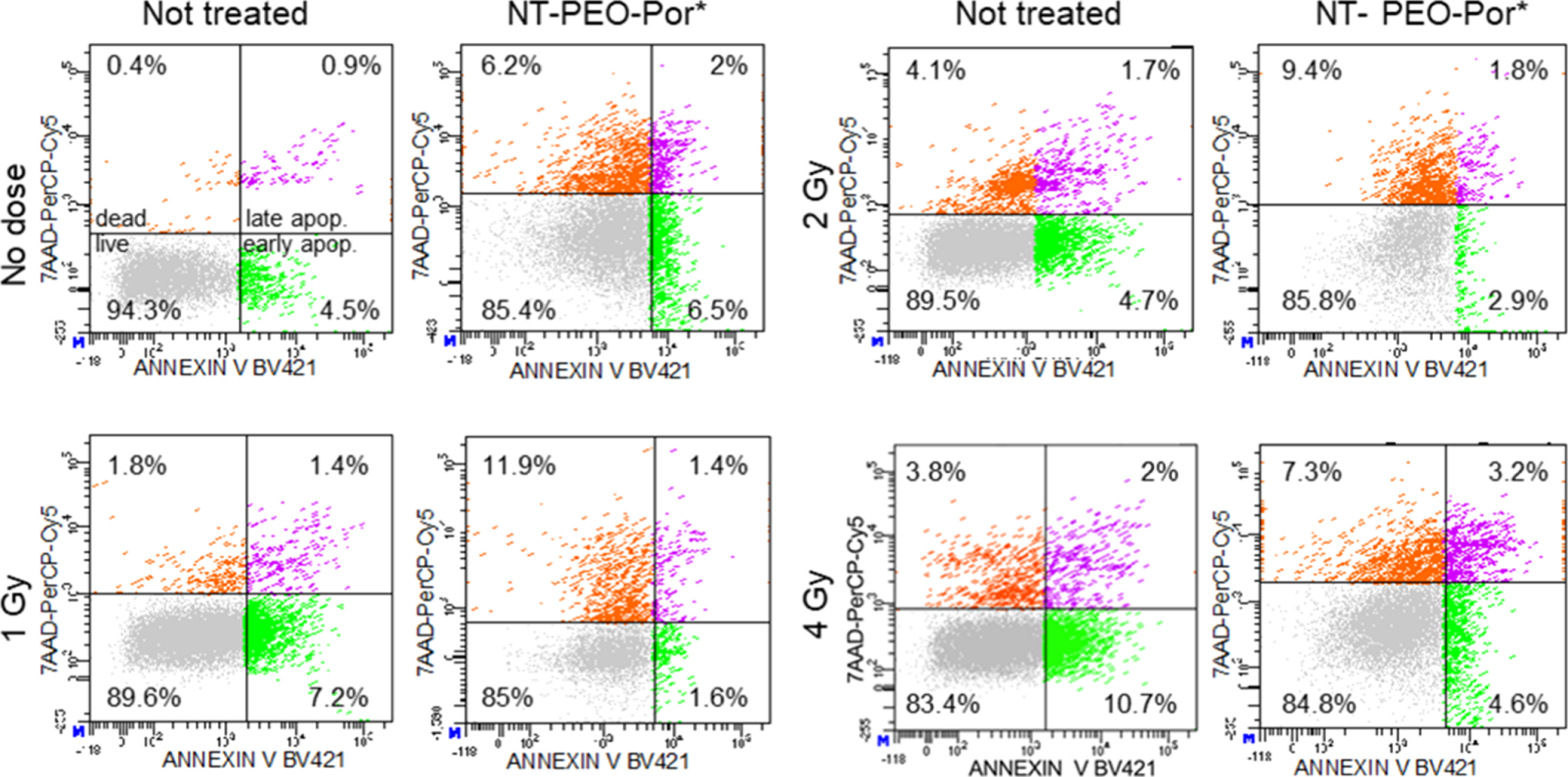
Representative dot charts of annexin-V/7-AAD bivariate flow cytometry. U-87 cells were treated with 20 μg cm^–2^ NT-PEO-Por* with or without radiation exposure (1, 2, and 4 Gy). Flow cytometry assay analysis was performed 4 h after X-ray exposure. The lower left quadrant (annexin_V–/7-AAD−) contains viable cells (gray). The lower right quadrant (annexin_V+/7-AAD−) represents early apoptotic cells (green). The upper left quadrant (7-AAD+/annexin V) shows necrotic cells (orange), and the right quadrant (7-AAD+/annexin_V+) shows late apoptotic cells (purple).

As anticipated above, the ionizing radiation is exploited in RT because of its ability to damage the cellular DNA and generate cytotoxic species. In both cases, the damaged and stressed cells automatically recognize the impossibility to survive and activate an irreversible programmed death mechanism named apoptosis.^61^ Evasion of programmed cell death is a hallmark of human cancer.^62^ The triggering of apoptosis is therefore crucial to further enhance the therapeutic efficiency of cancer treatments, in parallel to the prompt necrosis driven by hyperthermia. The occurrence of an apoptotic cell population has been monitored by flow cytometry experiments performed as a function of the delivered dose. As shown in Figure 5, the results obtained with experiments made 4 h after X-ray exposure confirm that the U-87 cells stained with NT-PEO-Por* undergo higher necrosis than unstained U-87, especially at 1 and 2 Gy of delivered dose where the necrotic cell subpopulation represented 11.9 and 9.4%, respectively. In line, the presence of NT-PEO-Por* pushed irradiated cells to rapidly activate the process of apoptosis, which at higher doses results into the prevalence of late-stage apoptotic events compared to unstained cells, at higher doses. Conversely, the unstained U-87 cells display a lower tendency toward necrotic events, according to the lower amount of energy deposited, while the apoptotic subpopulation percentages tends to a slow increase, particularly for the early apoptotic events. We note that a discrepancy between early and late apoptotic cell percentages at increasing doses may be caused by the short time frame of the early apoptotic phase, which can often be missed since cells are seen as either live or late apoptotic.

**Figure 6:**
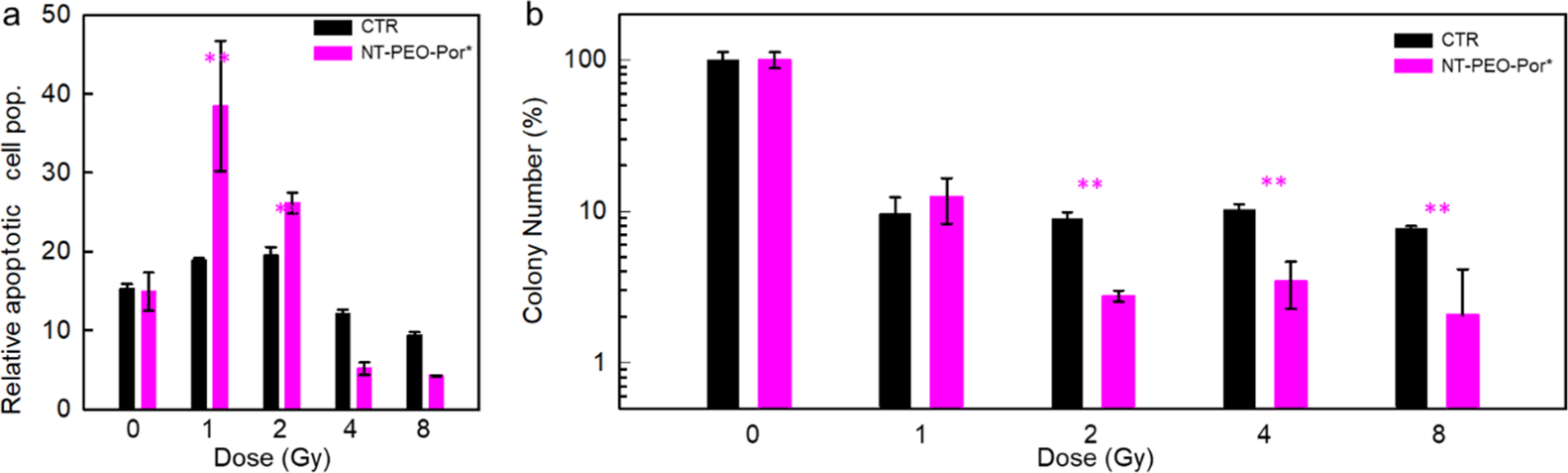
(a) Evaluation of apoptotic pathway activation by the Caspase-Glo 3/7 test on U-87 cells stained with 20 μg cm^–2^ of NT-PEO-Por* as a function of the nominal dose delivered, evaluated 24 h after X-ray exposure. (b) Evaluation of dose-dependent loss of clonogenic capacity by monitoring colony formation in U-87 cells stained with 20 μg cm^–2^ of NT-PEO-Por* as a function of the nominal dose delivered after 21 days from X-ray exposure. Statistical analyses: two-way ANOVA, *P* < 0.01**. Error bars are the standard deviations of the mean values calculated for five independent experiments.

Figure 6a reports the results of the specific apoptosis assay performed by measuring the caspase enzyme activity 24 h after radiation.^63^ The data show that functionalized NTs also strongly promote the activation of apoptosis at low doses. In particular, with 1 Gy of delivered dose, the number of apoptotic cells is doubled with respect to the control sample; moreover, by increasing the delivered dose to 2 Gy, we still observe an apoptosis sensitization effect in the stained sample. Further increasing the delivered dose makes the assay not affordable since, as discussed above, the fraction of surviving non-necrotic cells is too low. Overall, the results obtained indicate a prompt effect of the RPDT agents on necrosis and a subsequent effect on induced apoptosis of U-87 cells, suggesting a combination of factors involved in cell death, which can result in a therapeutic effect for a prolonged time after the treatment. This latter is evaluated by the colony formation assay (see the Experimental Section), which documents the clonogenic ability of the cells that survived and totally recovered from the damage due to X-ray exposure and therefore is generally taken as a model of the tumor response *in vivo*.^64^ Remarkably, U-87 cells are more responsive to NT-mediated growth inhibition (Figure 6b). The unstained colony under a 1 Gy exposure shows a reduction of the U-87 proliferation ability of about 90%, but no further change is observed for delivered doses up to 8 Gy. Conversely, the use of the RPDT agent induces a 97% reduction of the U-87 clonogenic ability with 2 Gy of delivered dose. This implies that we achieved an almost complete suppression of the recovering and reproduction ability of the treated cells, which strongly suggest additional beneficial effects of RPDT in the clinical perspective of scheduled spaced-out treatments of cancer patients.

## Conclusions

Recent research studies proved that RPDT is efficient in the treatments of oncological diseases. We developed here a multicomponent nanomaterial based on an inorganic dense nanoscintillator as an RT enhancer, coupled with a conjugated dye as a scintillation-activated sensitizer of the singlet oxygen cytotoxic species. In order to maximize the synergistic effect of the two components, the ROS-sensitization ability under exposure to ionizing radiation of each constituent has been studied. The results obtained demonstrate, as general guidelines for the development of such systems, that particular care should be taken with the PS condition once coupled to the nanoscintillator in order to avoid parasitic mechanisms that can limit its sensitization ability and therefore the overall system performance. On the other hand, in the optimized composition, the functionalized NTs show a significantly improved ability in the sensitization of the singlet oxygen under low irradiation doses with respect to the bare nanoscintillators. Thanks to their excellent biocompatibility, the functionalized NTs have been tested as RPDT agents in model human tumor cells. The enhancement of the therapeutic effect of the ionizing radiation exposure in the presence of functionalized NTs is evident. The series of in vitro assays performed indicates indeed that functionalized NTs, thanks to increased energy release given by their interaction with high-energy radiation and to the sensitized production of cytotoxic ROS, help to both (i) promptly kill the tumorigenic cells by boosting the thermal shock and (ii) limit their reproduction by favoring the triggering of the apoptosis mechanism. Considering the versatility of the synthetic protocol employed, the high biocompatibility, and the recently demonstrated ability of NTs in penetrating the blood brain barrier of brain cancer of murine models, these findings strongly support their future *in vivo* tests for the treatment on cerebral tumors.^38^ In addition, it is worth noting that the beneficial activity of the RPDT agent is particularly evident at low levels of delivered doses, which are strictly comparable to those employed in the hospitals for clinical treatments. These pieces of evidence suggest the possibility to effectively reduce the therapy exposure time and consequently limit the occurrence of collateral effects due to a prolonged exposure to high-energy radiation of the patients, with evident beneficial consequences on both the healthcare and socioeconomic perspectives.

## Methods

### Synthesis of Stoichiometric Chrysotile NTs

Chrysotile NTs were synthesized according to the modification of the synthesis method reported in the literature.^65^ A hydrothermal synthesis reactor with a 100 cm^3^ moveable polypropylene vessel was used to carry out the hydrothermal reaction of Na_2_SiO_3_ and MgCl_2_ in an aqueous NaOH (0.4 M) solution at 250 °C, on the saturated vapor pressure curve (39 atm), and with a run duration of 8 h. The precipitate removed from the solution was repeatedly washed with deionized water before being dried for 3 h at 110 °C.

### Synthesis of Functionalized NTs

Functionalized NTs were prepared in water in accordance with the ionic self-assembly procedure reported in De Luca et al.^44^ In a typical reaction batch, 40 mg of chrysotile was suspended in 8 mL of 5 × 10^–3^ M phosphate buffer at pH 7 and the PS solution (6.5 × 10^–4^ M) was added slowly under vigorous stirring. Note that RB requires a deprotonation procedure with NaOH in order to allow its solubilization in water. PS molecules adsorb on the surface of NTs, and after saturating it with ≈5 mL of the solution, they start to confer to the liquid a colorful appearance. The hybrid composite NTs were centrifuged and washed with water and finally dried in vacuum. Partial coverage of the NT surface with the PS is obtained by adding equal volumes of 1:50 diluted PS solution.

### Electronic Microscopy

Transmission electron microscopy (TEM) observations were performed with a JEOL JEM-1220 operating at 120 kV, which was used to image the NTs. TEM samples were prepared by dispersing a few milligrams of the compounds in 2 mL of distilled water and dropping 3 μL of the solution on carbon-coated copper grids.

### Diffraction Experiment

Powder X-ray diffraction (PXRD) was performed using an X’Pert Pro (Malvern Panalytical) powder X-ray diffractometer equipped with a Cu X-ray tube, which was used to collect diffraction patterns of the synthesized minerals and hybrid NTs.

### Synthesis of mPEG2K-phosphate

0.5 mmol of mPEG is dissolved in dry DCM and added dropwise to a solution of phosphoryl chloride (POCl_3_, 1.2 equiv) and tetraethylammonium (2.4 equiv) in an ice bath. The reaction mixture is slowly brought to room temperature and stirred for 24 h. Then, 5 mL of deionized water is slowly added to the mixture and left to react for 1 h. The solvent is removed in vacuum, and the crude product is dissolved in DCM and extracted with acidic water (HCl 0.2 mM) and then extracted three times with saturated brine. The organic phase is collected and dried over MgSO_3_, filtered, and precipitated three times in diethyl ether ^1^H NMR (500 MHz, CDCl_3_): 4.12 ppm (m, ^2^H, CH_2_CH_2_-O-P), δ 3.62 ppm (m, 454H, −CH_2_CH_2_– PEO chain), δ 3.34 ppm (s, ^3^H, −OCH_3_); ^31^P NMR (500 MHz, CDCl_3_): δ 2.01 ppm (s, ^1^P, OP(OH)_2_).

### Pegylation of Dye-Functionalized NTs

Partially covered NTs were dispersed in 100 mL of THF (0.5 mg mL^–1^) and sonicated until the solution became homogeneous. Then, 0.125 mmol of *m*PEG2K-phosphate was added in the solution, and the mixture was then refluxed overnight. The crude product was then centrifuged three times with THF in order to wash the NT from not reacted polymer chains. The fibers were then dried and stored in a fridge. The FT-IR spectrum shown in Fig. S4 demonstrates the successful grafting of PEO on the NT surfaces.

### X-ray Experiments

RL measurements were performed by irradiating the samples at room temperature with a Philips 2274 (steady-state RL spectroscopy and the singlet oxygen production monitoring experiment) or a Machlett OEG 50 (*in vitro* cell irradiation experiments) X-ray tube, both with a tungsten target, equipped with a beryllium window and operated at 20 kV X-rays. At low voltage, X-rays are generated only by the *bremsstrahlung* mechanism. No beam filtering has been applied. RL spectra have been recorded using a homemade apparatus featuring a liquid nitrogen-cooled charge-coupled device (CCD, Jobin-Yvon Spectrum One 3000) coupled to a monochromator (Jobin-Yvon Triax 180) with a 100 grooves/mm grating as a detection system. The spectra have been corrected for the setup optical response.

### Optical Studies

Steady-state PL and PLE spectra have been recorded using a xenon lamp as an excitation source, together with a double monochromator (Jobin-Yvon Gemini 180 with a 1200 grooves/mm grating), and recorded through a nitrogen-cooled CCD detector coupled to a monochromator (Jobin-Yvon Micro HR). Under *cw* laser excitation, signals have been recorded using a nitrogen-cooled CCD coupled with a double monochromator, Triax-190 (HORIBA Jobin-Yvon), with a spectral resolution of 0.5 nm. The PL quantum yield of bare NTs has been measured with relative methods using 2,5-diphenyloxazole as a fluorescence standard.^66^ All spectra have been corrected for the setup optical response. Time-resolved PL spectra have been recorded using a pulsed LED at 340 nm (3.65 eV, EP-LED 340 Edinburgh Instruments, a pulse width of 700 ps) or a pulsed laser at 405 nm (3.06 eV, EPL-405 Edinburgh Instruments, a pulse width of 150 ps) as a light source. Data were obtained with an Edinburgh Instruments FLS-980 spectrophotometer, with a 5 nm bandwidth and a time resolution of 0.1 ns.

### Singlet Oxygen Relative Concentration Measurement

The optical probe SOSG has been purchased from Thermo Fisher and used as is. The SOSG powered has been diluted in a 1:10 solution of dimethyl sulfoxide (DMSO) and PBS, which has been used to disperse the NTs. The intensity of the SOSG fluorescence, which is directly proportional to the concentration of singlet oxygen in the environment, has been monitored under *cw* laser light excitation at 473 nm (Figure S5).

### Cell Culture

Human primary glioblastoma cells U-87 were purchased from ATCC (HTB-14) and thawed in pre-warmed Dulbecco’s modified Eagle’s medium (DMEM, Gibco) supplemented with 15% fetal bovine serum (FBS, Gibco). Cells were seeded in a 75 cm^2^ flask and incubated at 37 °C and 5% CO_2_ until 90% of confluence was reached. The cell culture medium was changed every 2 days.

### Confocal Microscopy

U-87 cells were seeded onto rounded coverslips housed into 12-well plates at a density of 30.000 cells/cm^2^ in DMEM supplemented with 15% FBS and 50 μg/mL gentamicin (Gibco). After 24 h, 20, 40, and 60 μg cm^–2^ NT-PEO-Por* were added to cells and incubated overnight. Briefly, 25 mg/mL NT-PEO-Por* stock solution was prepared in distillated sterile water and sonicated by means of an ultrasound bath for 30 min to break big aggregates. NT-PEO-Por* dilutions were calculated accordingly to final concentrations and mixed directly into the complete cell medium. For fluorescence imaging, NT-PEO-Por*-labeled U-87 cells were washed twice with PBS (without Ca^2+^ and Mg^2+^, Gibco), fixed with 4% paraformaldehyde, and permeabilized with 0.1% Triton X-100 in PBS for 15 min. 1% BSA solution in PBS was then added for 45 min to reduce not specific background staining. Alexa Fluor 488 phalloidin (Thermo Fisher Scientific) was diluted in PBS according to the manufacturer’s instructions, added to cells, and incubated at room temperature for 45 min. Cells were then washed twice in PBS, and coverslips were removed from the multiwells and mounted onto glass slides with the Fluoromount-G medium (Thermo Fisher Scientific). Z-stack images were obtained with a confocal microscope Leica TCS SP8 with a white light laser.

### Cell Viability

For cell viability experiments, cells were seeded in 96-well plates at a density of 3 × 10^3^ cells/well (*n* = 6 for each condition); after 24 h, NT-PEO-Por* was added to the complete cell medium, as previously described. U-87 cells were washed twice in PBS, and the MTT test was performed (methylthiazolyldiphenyl-tetrazolium bromide, Sigma) at 24, 48, and 72 h from NT-PEO-Por* labeling according to the manufacturer’s instructions. Briefly, a 50 μg/mL MTT solution was added to the samples; after 3 h of incubation at 37 °C, the medium was removed, the converted dye was solubilized with DMSO (Sigma), and the absorbance was measured at 560 nm (GloMax Discover, Promega). Not labeled cells in the complete medium were used as control conditions.

### Viability Test under Irradiation (Trypan Blue Exclusion Assay and MTT)

U-87 cells were seeded in 35 mm cell culture dishes at a density of 3000 cells cm^–2^ in DMEM (Gibco) supplemented with 15% FBS and 50 μg/mL gentamicin (Gibco). 25 mg/mL NT-PEO and NT-PEO-Por* were resuspended in distillated sterile water and sonicated for 30 min. After 24 h from seeding, cells were treated with 20 μg cm^–2^ of NT-PEO or NT-PEO-Por* and incubated overnight. Untreated cells were used as the control. Cells were washed with PBS and after X-ray exposure at different doses (0, 1, 2, 4, 8, and 12 Gy) were detached by 0.25% trypsin–ethylenediaminetetraacetate (EDTA) (Thermo Fisher Scientific). In order to distinguish dead cells, 20 μL of a U-87 suspension was stained with an equal volume of Trypan blue 0.4% (Thermo Fisher Scientific) and counted using a Fast Read 102 chamber. The cell concentration was determined by the formula cells/mL = [(∑cells counted in 5 squares/5) × dilution factor × 10^4^]. The percentage of death cells was calculated by the following formula: % dead cells = (number of blue cells ÷ number of total cells) × 100. All the experiments were performed in triplicate. MTT assessment was performed as already described with minimum modifications. Immediately after X-ray exposure, the MTT solution was directly added to dishes housing the X-ray-treated cells and incubated for 3 h. In order to avoid the loss of stressed cells detaching from dishes during the assay, supernatants and cells were collected and centrifuged at 1200 rpm for 5 min before adding DMSO.

### Caspase 3/7 Activity Detection

For the evaluation of caspase, we performed the Caspase-Glo 3/7 assay (Promega), which measures caspase-3 and caspase-7 activities through a luminescence signal, following the manufacturer’s instructions. Briefly, unstained and NT-PEO-Por*-stained U-87 that were exposed to escalating doses of X-rays were detached by 0.25% trypsin–EDTA, counted, and seeded into a white-walled 96-well plate at 3000/cm^2^. Cells were then incubated in 100 μL of the fresh medium for 24 h to let them adhere. 100 μL of the Caspase-Glo 3/7 Reagent was then added to the medium and incubated for 3 h at 37 °C, and the luminescence was recorded with Glomax multiplate readers. The Caspase-Glo 3/7 Reagent was also added to the fresh medium without cells in order to measure the background luminescence. Before apoptosis evaluation, representative images of seeded cells were obtained using a Leica DMi8 inverted microscope.

### Flow Cytometry

The induction of apoptosis and necrosis of U-87 was measured 4 h after X-ray exposure by flow cytometry. X-ray-exposed unstained and NT-PEO-Por*-stained U-87 were detached from dishes by 0.25% trypsin–EDTA, centrifuged at 1200 rpm for 5 min, and resuspended in 100 μL of the annexin buffer (BD) and 5 μL of Annexin V BV421 (BD), which binds to phosphatidylserin (PS) residues and allows the identification of apoptotic cells. Cells were incubated a 4 °C for 20 min, washed in the annexin buffer, and centrifuged. The viability dye 7-aminoactinomycin D (7-AAD, BD) was added to cell pellets to stain non-viable cells and recognize late-apoptotic fractions. For fluorescence-activated cell sorting (FACS) characterization, data were obtained using a FACSAria Fusion cell sorter, equipped with five lasers, and analyzed with FACSDiva software (ver. 8.0, BD). At least 10 × 10^3^ events were recorded for each condition. Debris events were excluded from the analysis by morphological gating (side scatter vs forward scatter dot plot).

## Supporting Information Available

RL emission band assignment discussion, time-resolved data analysis, FT-IR data, and singlet oxygen measurement raw data (PDF)
