# Supplementary material for: Functionalized Scintillating Nanotubes for Simultaneous Radio- and Photodynamic Therapy of Cancer
Source: ACS Appl Mater Interfaces. 2021 Mar 15;13(11):12997–3008. doi: 10.1021/acsami.1c02504 (PMC8153399; doi:10.1021/acsami.1c02504)
Supplement: Supplementary file 1 — am1c02504_si_001.pdf [file am1c02504_si_001.pdf]

Supporting Information for

Functionalized scintillating nanotubes for simultaneous radio and photodynamic therapy of cancer.

*Irene Villa[1‡], Chiara Villa[2‡], Roberta Crapanzano[1], Valeria Secchi[1], Massimo Tawfilas[1], Elena Trombetta[4], Laura Porretti[4], Andrea Brambilla[2], Marcello Campione[3], Yvan Torrente[2], Anna Vedda[1], Angelo Monguzzi[1]**

[1]Dipartimento di Scienza dei Materiali, Università degli Studi Milano-Bicocca, via R. Cozzi 55, 20125 Milano, Italy.

[2]Stem Cell Laboratory, Department of Pathophysiology and Transplantation, Università degli Studi di Milano, Fondazione IRCCS Ca' Granda Ospedale Maggiore Policlinico, Centro Dino Ferrari, via F. Sforza 35, 20122 Milan, Italy.

[3]Dipartimento di Scienze dell'Ambiente e della Terra Università degli Studi Milano-Bicocca, Piazza della Scienza, 20125 Milano, Italy.

[4]Servizio di Citofluorimetria, Laboratorio Analisi, Fondazione IRCCS Ca' Granda Ospedale Maggiore Policlinico, via F. Sforza 35, 20122 Milan, Italy.

**E-mail: angelo.monguzzi@unimib.it*

Characterization of functionalized nanotubes.

Functionalized nanotubes NT-ErB, NT-RB, and NT-Por, synthesized as described in the main text, are studied by optical absorption experiments. Fig. S1 shows the absorption spectrum of dispersions of functionalized NTs in PBS at normal incidence in a quartz cuvette with optical path of 1 cm. The absorbance of the dyes is calculated by subtracting the scattering background due to NTs at the absorption peak. By considering the molar extinction coefficient ε to the employed photosensitizers, we therefore calculate the number of dyes in the dispersion and their total weight, which results negligible. Therefore, given the chrysotile density of 2.53 g/cm^3, a tube geometry with average length 58 nm, inner diameter 7 nm, and outer diameter 20 nm, we can calculate the number of NTs in the dispersion and therefore the average number of photosensitizers anchored to the NTs surfaces. Table T1 reports the results obtained for each sample.

For the optimized NT-PEO-Por* sample, thermogravimetric analysis (TGA) was performed to evaluate the percentage of the organic compound tethered onto the NT surface. The data in Fig. S6 indicates that that the PEO accounts for the 6% of the total weight. The amount of dye in this composition cannot be evaluated by this technique due to sensitivity limit.

Table T1. Peak absorbance value, molar extinction coefficient and calculated number of dye per NT from the absorption measurements in Fig. S1.

	composition	**A**	ε [cm^{-1} M^{-1}]	**Dyes weight** [mg]	**#dye/NT**
sample					
**NT-ErB**	1.3mg/3mL	0.14	82500	2.2×10^{-3}	90
**NT-RB**	1.25mg/3mL	0.41	65786	1.8×10^{-2}	378
**NT-Por**	0.4mg/1mL	0.21	295013	2.2×10^{-3}	467

RL band assignment. In the literature, the chrysotile luminescence properties lying in the 460-480 nm range are compared to the emissions in silica and alumina solids and associated with OH- radical groups [1] The NT luminescence band at 530 nm is tentatively associated to oxygen defects within the lattice [2] In addition, in insulating materials, the presence of a luminescence at energies below the bang gap is ascribed to the formation of self-trapped excitons (STE). [3] The controversy of the PL and RL luminescence origin in natural chrysotile NT is mostly related to the presence of several contaminants, such as other minerals and trace metals (Al, Fe, Mn…), that are particularly abundant in chrysotile lattice, resulting in a large variety of structural defects, isomorphic substitutions, and impurities.[4] Especially, luminescent components at wavelengths longer than 520 nm may be related to trace metals [5] [6] [7]. Synthesized and natural chrysotile can be also mixed with other minerals, which remain as contaminants. This is the case of brucite, $Mg(OH)_2$, found in the NT synthesized in this work that is responsible for the 750 nm luminescence [8].

Time resolved data analysis. The evolution of the PL intensity decay in time $I_{PL}(t)$ has been reproduced with an analytically multi-exponential function.

$$I_{PL}(t) \propto \sum_1^i A_i e^{-\left(t/\tau_i\right)}. \qquad \text{Eq. S1}$$

The parameters used for the fitting procedure are reported below in Tabs. 1 and 2. The average PL lifetime for the nanotubes PL discussed in the min text has been calculated as the weighted average of the characteristic decay time for each exponential function i using

$$\tau_{NT} = \frac{\sum_1^i A_i \tau_i}{\sum_1^i A_i}. \quad \text{Eq. S2}$$

Table T2. Fit parameters for the dyes (Erythrosine B, ErB - Rose Bengal, RB – HTPPS^{4-}porphyrin, Por) and functionalized nanotubes (NT- ErB, NT-RB, NT-Por) emission decay, recorded at the maximum of the PL spectrum (Fig. 2b).

	**A_1**	**τ_1** (ns)	**A_2**	**τ_2** (ns)	**A_3**	**τ_3** (ns)
**ErB**	0.99	0.7	0.01	10.7	-	-
**NT-ErB**	0.93	0.4	0.07	4.7	-	-
**RB**	1.0	0.9	-	-	-	-
**NT-RB**	0.82	0.7	0.13	2.9	0.05	26.5
**Por**	0.40	1.8	0.60	10.10	-	-
**NT-Por**	0.71	1.3	0.29	9.2	-	-
**NT-PEO-Por***	0.48	3.0	0.52	10.2		

Table T3. Parameters for the nanotubes PL decay at 420 nm (Fig.3b) as a function of the dye species attached on the surfaces, specifically Erythrosine B (NT- ErB), Rose Bengal (NT-RB) and HTPPS^{4-}porphyrin (NT-Por).

	**A_1**	**τ_1** (ps)	**A_2**	**τ_2** (ps)
**Nanotube**	0.25	189	0.75	828
**NT-ErB**	0.67	45	0.33	638
**NT-RB**	0.54	48	0.46	701
**NT-Por**	0.97	10	0.03	689

Table T4. Net efficiency of the ET yield $\overline{\Phi}_{ET}$ form NT to surface dyes calculated form as the ratio between the integral of the blue PL intensity decay in presence (I_{dye}) and in absence (I_{NT}) of anchored chromophores (Fig.3b of the main text).

$$\overline{\Phi}_{ET} = 1 - \frac{I_{dye}}{I_{NT}}$$

	$\overline{\Phi}_{ET}$
**NT-ErB**	0.45
**NT-RB**	0.63
**NT-Por**	0.83

Supplementary Data

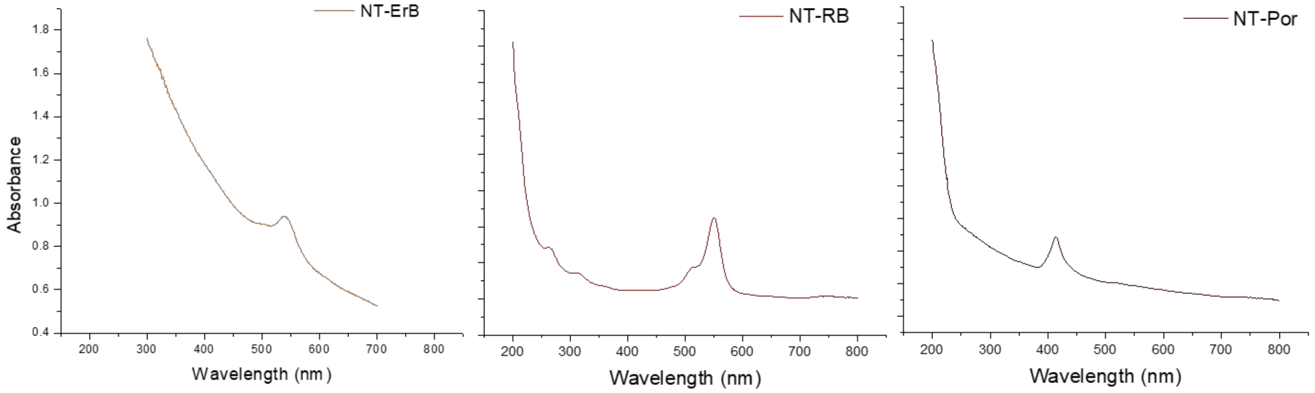

Fig. S1. Absorption spectra of functionalized NTs dispersions NT-ErB (1.3mg/3mL), NT-RB (1.25mg/3mL) and NT-Por (0.4mg/1mL) in PBS recorded at normal incidence in quartz Suprasil cuvettes with optical path 1 cm.

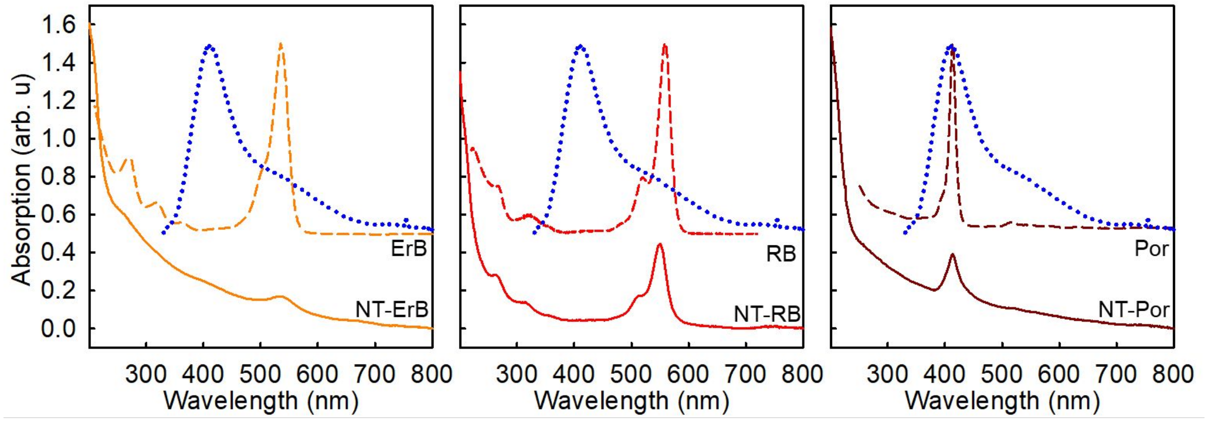

Fig. S2. Absorption spectra of photosensitizers (dashed lines) Erythrosine B (ErB), Rose Bengal (RB) and HTPPS4-porphyrin (Por) and functionalized nanotubes (solid lines, NT-ErB, NT-RB and NT-Por) in PBS. For reference the dotted line shoe the radioluminescence spectrum of bare NT to point out the resonance with the dyes absorption that enable ET from NT to photosensitizers on surfaces.

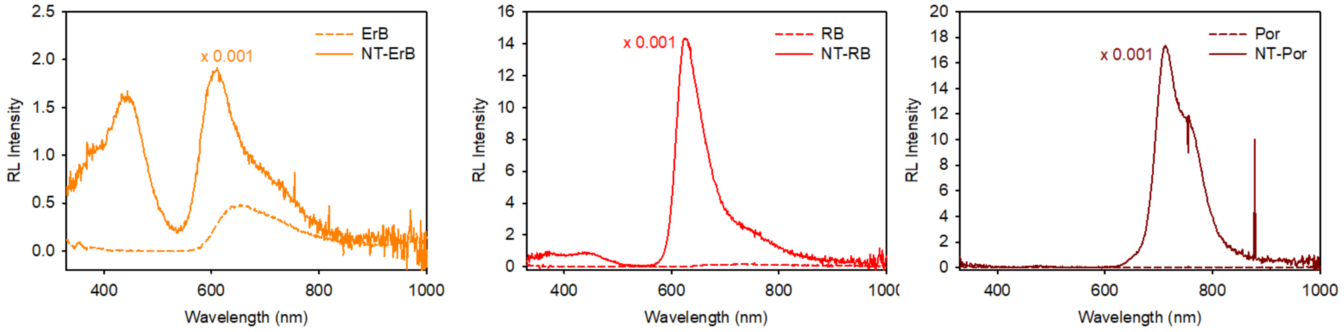

Fig. S3. Radioluminescence (RL) spectrum of photosensitizers Erythrosine B (ErB), Rose Bengal (RB) and HTPPS4-porphyrin (Por) and functionalized nanotubes (NT-ErB, NT-RB and NT-Por) as powder samples.

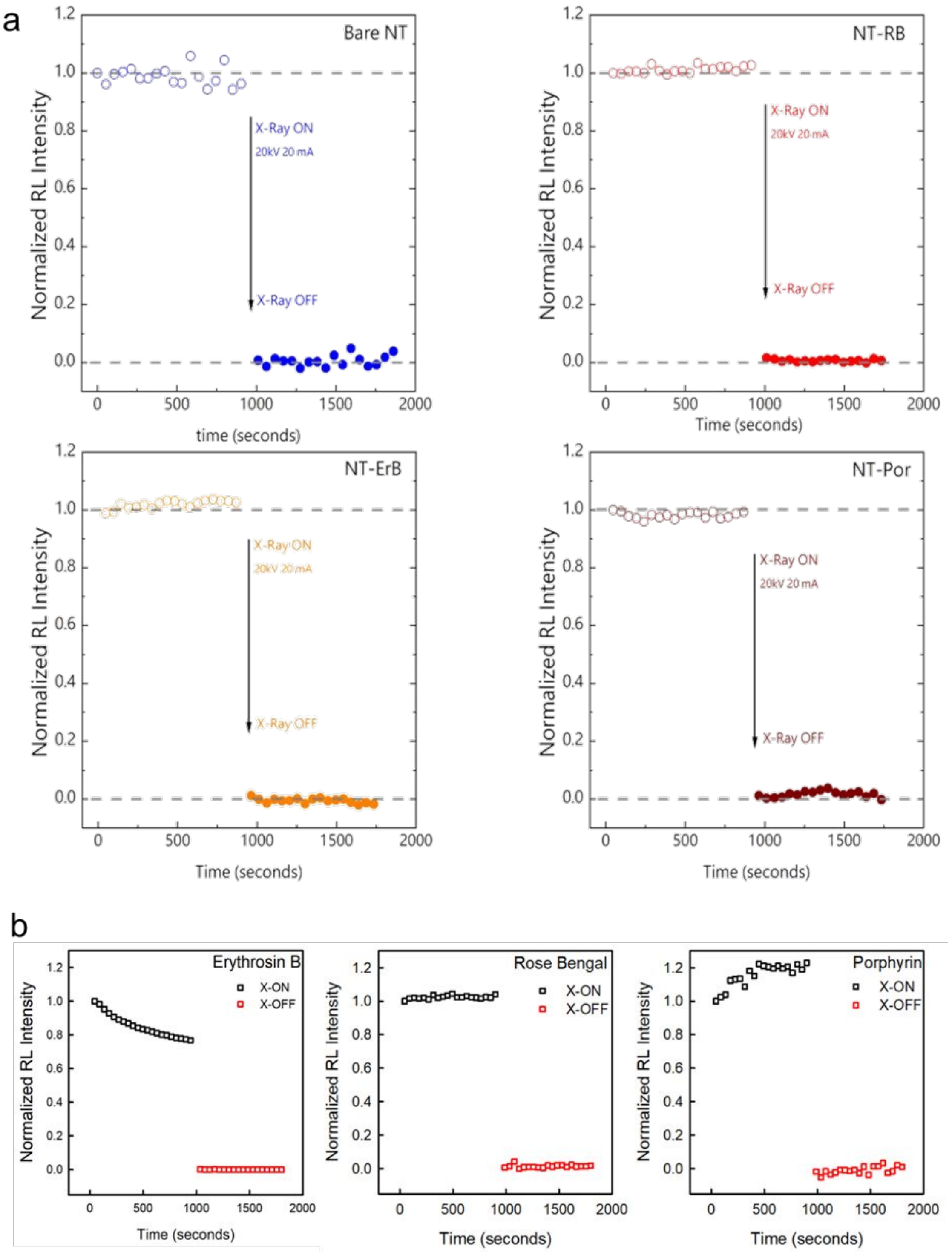

Fig. S4. (a) Normalized integrated RL intensity of bare and functionalized chrysotile as a function of irradiation time. NT have been excited under continuous X-ray irradiation in air (open dots) with a total accumulated dose of 200 Gy. RL signal is stable during the whole irradiation interval. In the Figure, dotted grey lines are guides for the eye. (b) Normalized integrated RL intensity of Erhytrosin B, Rose Bengal and Porphyrin as a function of irradiation time. Photosensitizers have been excited under continuous X-ray irradiation in air (open black squares) with a total accumulated dose of about 200 Gy. RL signal is stable during the whole irradiation interval. RL signal has been collected also after the shutdown of the irradiation (open red squares) suggesting the absence of delayed luminescence.

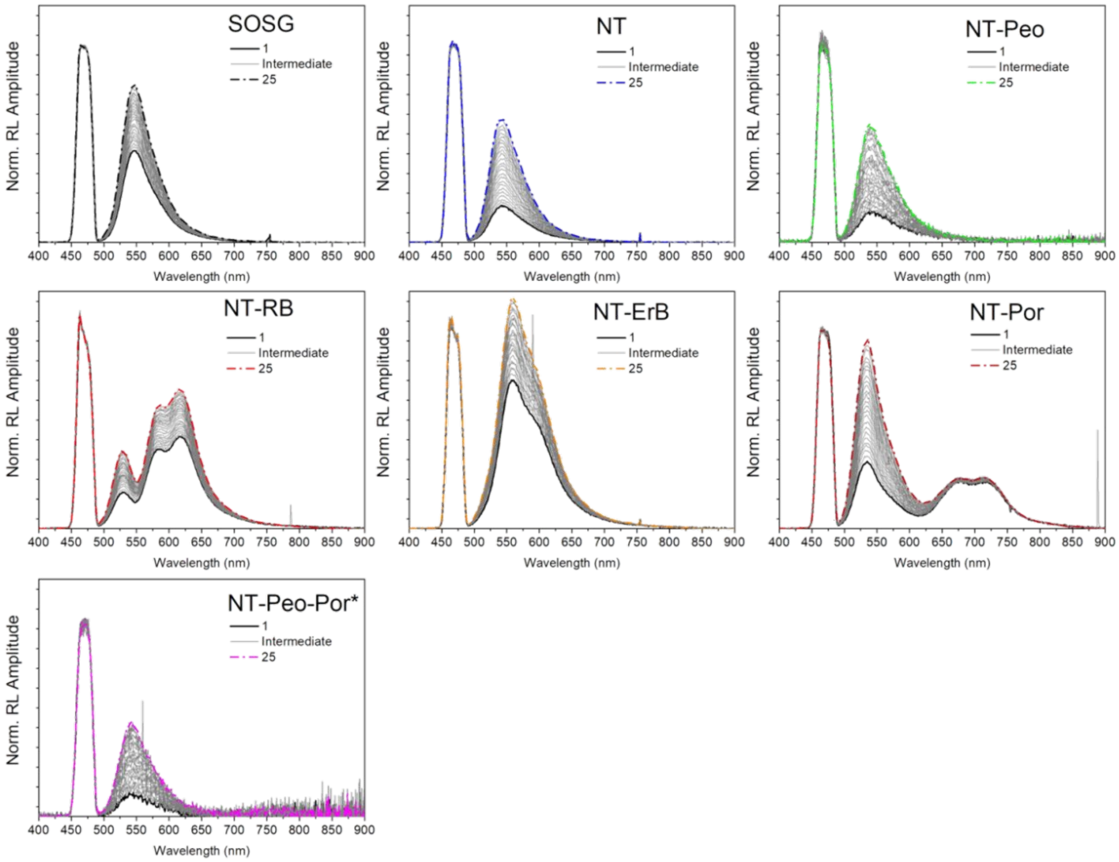

Fig. S5. Normalized RL spectra of all the NT-based systems. Full black lines correspond to the first (1) measurements; grey full lines are the intermediates spectra; and dotted lines correspond to the final (25) measurements. Bare NT and functionalized NT have been added into a solution of SOSG (SOSG powder in 1:10 solution of DMSO and PBS). X-ray tube voltage of 20 kV has been applied. The estimated dose for each measurement is of 4 Gy (nominal dose in air, calibrated on grinded crystalline quartz).

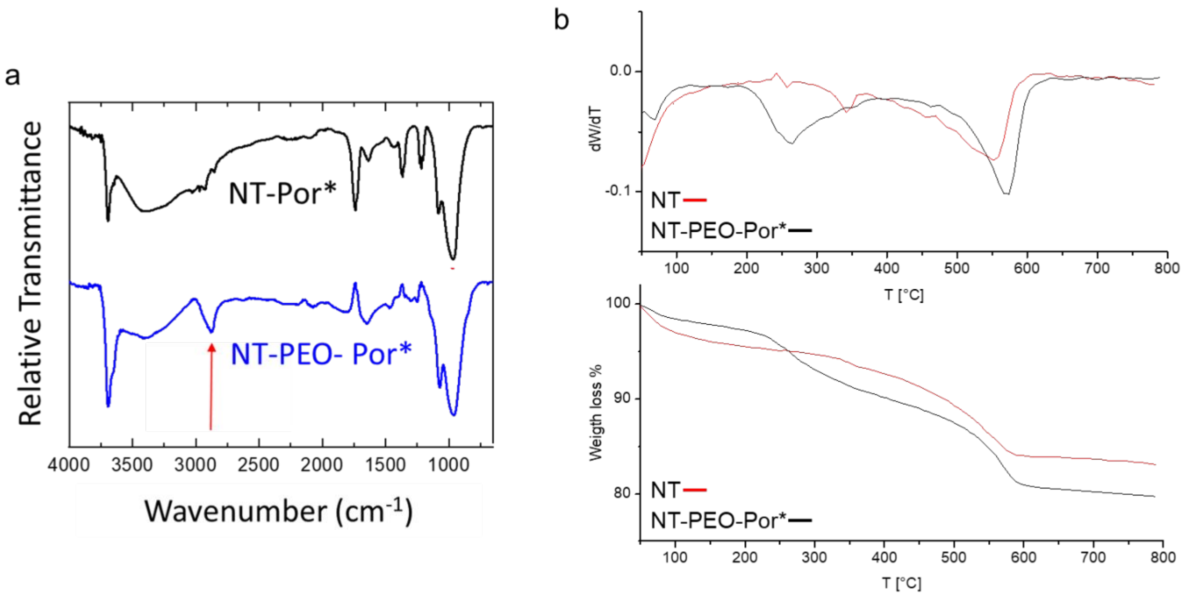

Fig. S6 – (a) Infra-red spectra clearly show the appearance of the CH_2-CH_2O vibrational peak of PEG chains around 2750-3000 cm^{-1} in the fluorescent nanotubes stabilized with PEO (NT-PEO-Por*) grafted on its surfaces, which is absent in the nanotubes functionalized only with porphyrins (NT-Por*). [9] (b) TGA analysis of sample NT-PEO-Por* compared to bare NT (Ref. 39 in the main text). In the multicomponent system it is clear visible the characteristic decomposition peak of PEO at around 250 °C [10].

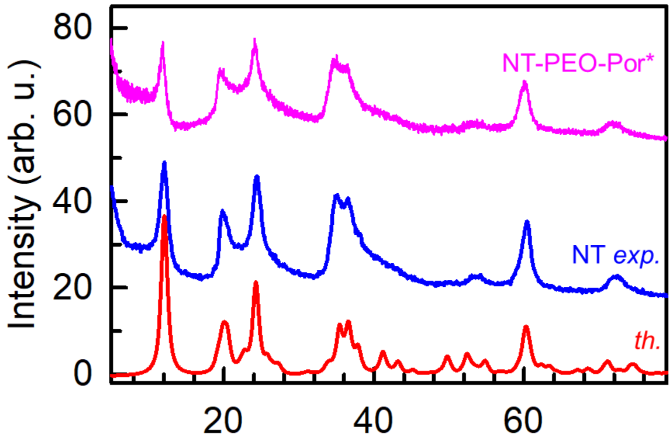

Fig. S7 - Powder X-ray diffraction (XRD) patterns of a reference chrysotile sample (*th.*) and of bare nanotubes as synthesized (NT *exp.*) and nanotubes functionalized with PEO as porphyrin (NT-PEO-Por*) as described in the main text.

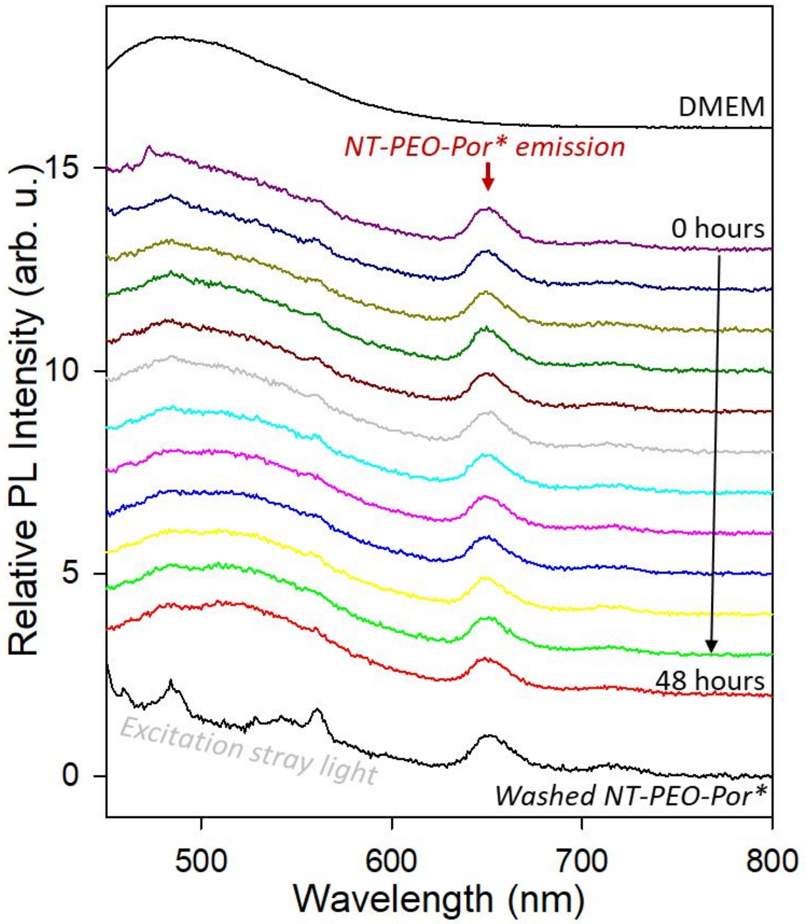

Fig. S8– Photoluminescence spectrum of a NT-PEO-Por* dispersion as a function of time under *cw* excitation at 410 nm. The dispersion has been then filtered to recover the nanotubes are dispersed in PBS for comparison. None change is observed in the NTs emission after washing with water, demonstrating the DMEM medium employed for cell growth does not affect the stability of the NTs functionalization with the luminescent porphyrin.

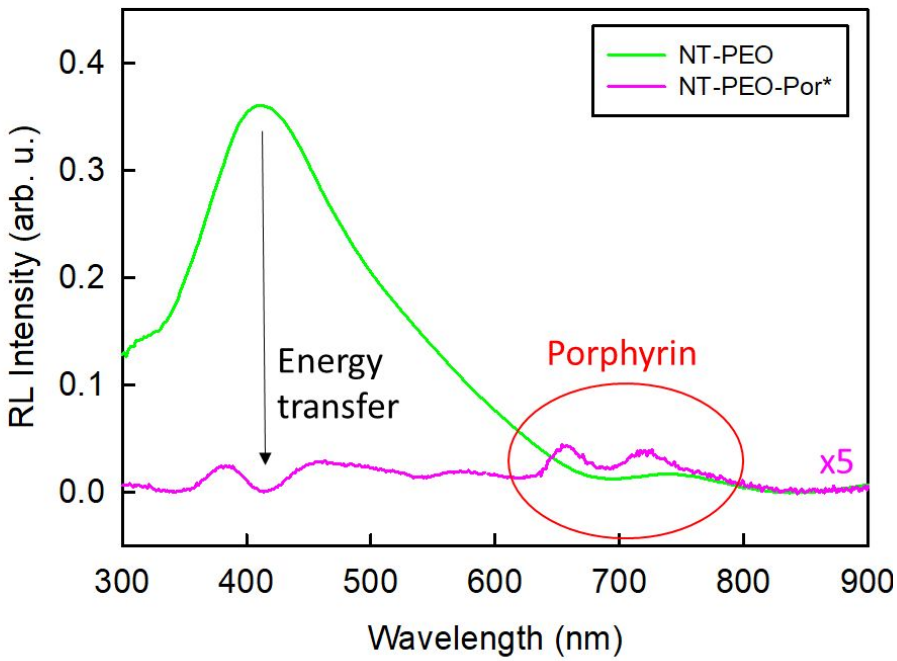

Fig. S9. Normalized RL spectra of all the NT-PEO and NT-PEO-Por* powders. X-ray tube voltage of 20 kV has been applied. The estimated dose for each measurement is of 4 Gy (nominal dose in air, calibrated on grinded crystalline quartz).

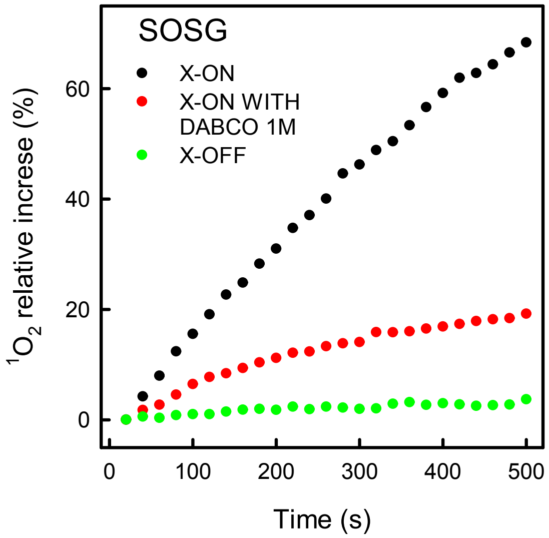

Fig. S10. Validation of the selectivity of SOSG probe to single oxygen. Measurement of the relative singlet oxygen concentration in PBS dispersions of SOSG as function of time (under X-rays exposure) (black dots). Comparison of the $^1O_2$ increment in the presence of singlet oxygen scavenger 1,4-diazobicyclo(2,2,2)-octane (DABCO, 1M) under irradiation, and after the shutdown of external excitation (red and green dots, respectively). All the measurements have been performed in air. The addition of the $^1O_2$ scavenger in the solution reduces the SOSG luminescence enhancement, suggesting that the SOSG increase is mostly due to the singlet oxygen moiety formation during the irradiation by x-rays in the ambient atmosphere (air) of the aqueous samples.

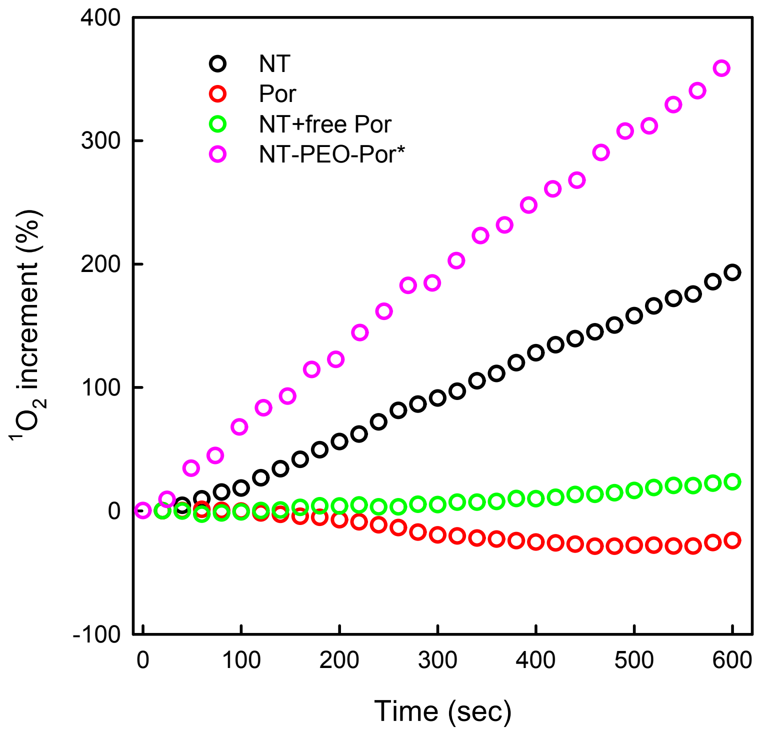

Fig. S11. Measurement of the relative singlet oxygen concentration in PBS dispersions of bare nanotubes (NT), free porphyrins (free Por) and mixed NT free porphyrin solution (NT+free Por) as a function of time under soft X-rays exposure.

Supplementary References

[1] S. Volkovetskii, A. Aksyuk, B. Shul'Gin, A. Shalyapin, *JApSp* **1973**, 18, 669.

[2] M. Bardosova, S. Romanov, C. S. Torres, N. Gaponik, A. Eychmueller, Y. Kumzerov, *Physica E: Low-dimensional Systems and Nanostructures* **2007**, 37, 218.

[3] D. M. Ramo, P. Sushko, A. Shluger, *Physical Review B* **2012**, 85, 024120.

[4] A. Bloise, D. Barca, A. F. Gualtieri, S. Pollastri, E. Belluso, *Environmental Pollution* **2016**, 216, 314.

[5] F. Pierini, E. Foresti, G. Fracasso, I. G. Lesci, N. Roveri, *Israel Journal of Chemistry* **2010**, 50, 484.

[6] S. Romanov, C. Sotomayor Torres, H. Yates, M. Pemble, V. Butko, V. Tretijakov, *Journal of applied physics* **1997**, 82, 380.

[7] V. Correcher, Y. Rodriguez-Lazcano, R. Gomesdarocha, J. García-Guinea, *Journal of Radioanalytical and Nuclear Chemistry* **2016**, 307, 1287.

[8] J. M. G. Davis, J. Addison, R. E. Bolton, K. Donaldson, A. D. Jones, B. G. Miller, *Carcinogenesis* **1985**, 6, 667.

[9] D. Selli, M. Tawfilas, M. Mauri, R. Simonutti, C. Di Valentin, *Chemistry of Materials* **2019**, 31, 7531.

[10] S. Ibrahim, M. R. Johan, *Int J Electrochem Sci* **2012**, 7, 2596.
